# Expression of cell cycle regulators and frequency of *TP53* mutations in high risk gastrointestinal stromal tumors prior to adjuvant imatinib treatment

**DOI:** 10.1371/journal.pone.0193048

**Published:** 2018-02-16

**Authors:** Michaela Angelika Ihle, Sebastian Huss, Wiebke Jeske, Wolfgang Hartmann, Sabine Merkelbach-Bruse, Hans-Ulrich Schildhaus, Reinhard Büttner, Harri Sihto, Kirsten Sundby Hall, Mikael Eriksson, Peter Reichardt, Heikki Joensuu, Eva Wardelmann

**Affiliations:** 1 Institute of Pathology, University Hospital Cologne, Cologne, Germany; 2 Gerhard Domagk Institute of Pathology, University Hospital Münster, Münster, Germany; 3 Institute of Pathology, University Hospital Göttingen, Göttingen, Germany; 4 Laboratory of Molecular Oncology, Translational Cancer Biology Program, University of Helsinki, Helsinki, Finland; 5 Department of Oncology, the Norwegian Radium Hospital, Oslo University Hospital, Oslo, Norway; 6 Department of Oncology, Skåne University Hospital, Lund University, Lund, Sweden; 7 HELIOS Klinikum Berlin-Buch, Berlin, Germany; 8 Department of Oncology, Helsinki University Hospital and University of Helsinki, Helsinki, Finland; University of Pittsburgh Cancer Institute, UNITED STATES

## Abstract

Despite of multitude investigations no reliable prognostic immunohistochemical biomarkers in GIST have been established so far with added value to predict the recurrence risk of high risk GIST besides mitotic count, primary location and size. In this study, we analyzed the prognostic relevance of eight cell cycle and apoptosis modulators and of TP53 mutations for prognosis in GIST with high risk of recurrence prior to adjuvant treatment with imatinib. In total, 400 patients with high risk for GIST recurrence were randomly assigned for adjuvant imatinib either for one or for three years following laparotomy. 320 primary tumor samples with available tumor tissue were immunohistochemically analyzed prior to treatment for the expression of cell cycle regulators and apoptosis modulators cyclin D1, p21, p16, CDK4, E2F1, MDM2, p53 and p-RB1. *TP53* mutational analysis was possible in 245 cases. A high expression of CDK4 was observed in 32.8% of all cases and was associated with a favorable recurrence free survival (RFS), whereas high expression of MDM2 (12.2%) or p53 (35.3%) was associated with a shorter RFS. These results were independent from the primary *KIT* or *PDGFRA* mutation. In GISTs with higher mitotic counts was a significantly increased expression of cyclin D1, p53 and E2F1. The expression of p16 and E2F1 significantly correlated to a non-gastric localization. Furthermore, we observed a significant higher expression of p21 and E2F1 in *KIT* mutant GISTs compared to *PDGFRA* mutant and wt GISTs. The overall frequency of *TP53* mutations was low (n = 8; 3.5%) and could not be predicted by the immunohistochemical expression of p53. In summary, mutation analysis in *TP53* plays a minor role in the subgroup of high-risk GIST before adjuvant treatment with imatinib. Strong expression of MDM2 and p53 correlated with a shorter recurrence free survival, whereas a strong expression of CDK4 correlated to a better recurrence free survival.

## Introduction

95% of gastrointestinal stromal tumors (GIST) exhibit an overexpression of the receptor tyrosine kinase KIT [1]. Approximately 90% harbor a mutation in the *KIT* gene or in the gene of the homolog receptor tyrosine kinase *PDGFRA*. These mutations lead to ligand independent, constitutive activation of the kinases and play a central role in GIST development [2]. A minority of GIST without gain-of-function mutations in one of these two kinases display other genetic and epigenetic changes [3–5]. These alterations are located in the *BRAF* (*rapidly accelerated fibrosarcoma B*) gene, in the *succinate dehydrogenase (SDH) complex* or the *insulin-like growth factor 1 receptor (IGF1R)* gene [6]. Furthermore, Hur *et al*. showed that especially genes of the cell cycle control are altered in high risk compared to low risk GIST [7]. One such gene is *TP53* encoding a 64 kDa protein (p53). Its gene product p53 is an essential regulator of many genes controlling apoptosis, senescence, DNA damage repair and cell cycle arrest [1]. Upon radiation-induced damage, p53 is activated as an element of the G1-phase checkpoint to allow DNA repair. Activation of p53 leads to the induction of p21 (CDKN2A) that binds to and inactivates cyclin-dependent kinases (CDK) complexed with cyclins’ (cyclin = CCN: cyclin D1/CDK4, cyclin D1/CDK6 and cyclin E1/CDK2). Consequently, the cell cycle is arrested [8]. Without this p21-induced inhibition complexed CDKs and cyclins phosphorylate the retinoblastoma 1 (RB1) protein that dissociates from E2F1 transcription factor. As a result transcriptional activation of a series of target genes leads to the progression of the cell cycle.

The frequency of *TP53* alterations is heterogeneous among different tumor entities. Less than 5% of cervical carcinoma harbor a *TP53* mutation whereas in ovarian carcinoma the *TP53* gene is altered in up to 90% [9]. Most of the mutations result in non-functional proteins destroying the normal tumor suppressing functions of p53 (TP53). Consequently, mutations in this gene are associated with accelerated tumor formation, higher stage, poor prognosis and low response to therapy [10–13]. A targeted therapy against mutated *TP53* is under development and some inhibitors are currently investigated in clinical trials [14–16]. Alternatively, loss-of-function mutations of *TP53* can be inactivated by other regulators of the cell cycle that convey p53 downstream effects as shown for MDM2 (*mouse double minute 2*) [11] and for CDK4 (*cyclin dependent kinase 4*) [17]. MDM2 binds either to the p53 transactivation domain to interfere with p53 transcriptional regulatory mechanism or promotes p53 proteasomal degradation by its ubiquitin ligase activity [18]. So the mechanisms how the tumor suppressor function of p53 is disturbed are diverse and the expression level of different cell cycle regulators in GIST may allow a deeper understanding of the p53 activation or inactivation status besides inactivating by *TP53* mutations. Targeted therapy against mutated p53 is under development and some inhibitors are currently investigated in clinical trials [14–16].

Another potentially relevant mechanism of GIST progression towards a more aggressive biological behaviour is a partial deletion of chromosome 9 playing an essential role in the transformation from a low to a high risk GIST [19, 20]. One possible result of this deletion is a p16 (CDKN2A) loss which normally functions as an inhibitor of the cell cycle. Inactivating mutations in this gene or its deletion seem to be essential to increase cell cycle activity and consequently to progress from low to high risk GIST.

In this study, we examined the *TP53* mutation frequency as well as the expression of clinically relevant cell cycle regulators and apoptosis modulators that are associated with impaired p53 function in 320 high risk GIST prior to adjuvant imatinib treatment cyclin D1 (CCND1), CDK4, p21 (CDKN1A), p16 (CDKN2A), E2F1, MDM2, p-RB1 and p53.

## Materials and methods

### Patient samples

Between February 2004 and September 2008, 400 patients with a high-risk GIST were included to the randomized, multicenter Scandinavian Sarcoma Group (SSG) trial XVIII, carried out in collaboration with the SSG and the German Working Group of Medical Oncology [Arbeitsgemeinschaft Internistische Onkologie (AIO)]. The study, a protocol-based pre-specified analysis, was approved by the Operative Ethics Committee of the Helsinki University Central Hospital (identifier NCT00116935).

Furthermore, ethical approvals were given from all local ethical committees of the other participating centers in Finland, Sweden, Norway and Germany. Before entering the study, the participants signed a consent form and gave their agreement to the study. All data were de-identified. In the study, patients who were diagnosed with gastrointestinal stromal tumor (GIST) were randomly allocated in a 1:1 ratio to receive imatinib (Gleevec) either for 12 or for 36 months following surgery. The participants of the study had to have a histologically verified GIST with a high risk of GIST recurrence despite complete removal of all macroscopic GIST tissue at surgery.

A high risk for GIST recurrence was defined by the presence of at least one of the following features: 1) the longest tumor diameter >10.0 cm, 2) tumor mitotic count >10 per 50 high power fields of the microscope, 3) tumor diameter > 5.0 cm and mitotic count >5, or 4) tumor rupture before surgery or at surgery. Two senior pathologists (E. W. and M. S.-R.) reviewed the histological diagnoses and carried out mitotic counting as described in a previous study [21]. Tissue from 320 primary GISTs, confirmed centrally to be GISTs, was available for the current study. Immunohistochemistry for CD34, KIT, PDGFRA, DOG1 and KI67 was carried out as described earlier [22, 23].

Mutation analyses were performed in 245 cases using Sanger sequencing for *KIT* exons 8, 9, 11, 13, 14, 15, 17 and *PDGFRA* exons 12, 14 and 18. Risk classification was done according to the modified National Cancer Institute (NIH) scheme [24].

### Immunohistochemistry

Immunohistochemical staining of cyclin D1 (also known as CCND1), CDK4, p21 (also known as CDKN1A), p16 (also known as CDKN2A), E2F1, MDM2, p-RB1 and p53 (TP53) was performed on an automatic stainer (medac GmbH, Wedel, Germany) using tissue microarrays (TMA) containing formalin-fixed paraffin-embedded tumor cores in duplicates. In brief, 3 μm sections were mounted on positively charged, adhesive slides and dried at 37°C overnight. For standard heat-induced epitope retrieval sections were boiled in citrate buffer pH 6 in case of CDK4, p21 (CDKN1A), E2F1, p-RB1 and p53 (TP53) or in EDTA buffer pH 6 in case of cyclin D1 (CCDN1), p16 (CDKN2A), MDM2 in a microwave oven. Primary antibody incubation was performed for 30 min at room temperature with appropriate antibodies (S1 Table).

The sections were subsequently incubated with polymer Poly-HRP-GAM/R/RIgG for 15 min followed by incubation for 8 min with 3.3’-diaminobenzidine (DAB) and washed. Slides were counterstained with hematoxylin.

Immunohistochemical score was adapted from Romeo et al. [25] and defined as follows: staining in 0–10% of tumor cells: score 0; 11–20%: score 1; 21–30%: score 2; 31–40%: score 3; 41–50%: score 4; 51–60%: score 5, 61–70%: score 6, 71–80%: score 7, 81–90%: score 8 and 91–100%: score 9. The intensity score was divided up in no visible staining: score 0, weak staining: score 1, moderate staining: score 2 and strong staining: score 3. Mean value of the sum and intensity score was built up as an overall score for the final analysis A mean score of >0 was regarded as high expression for cyclin D1 (CCND1), MDM2, CDK4 and p-RB1, one of >2 for p16 (CDKN2A), p53 (TP53) and E2F1 and one of >3 for p21 (CDKN1A).

### DNA isolation

In our cohort of 320 samples, 245 cases could be used for complete DNA isolation and sequencing analysis of *TP53* (76.6%). 75 samples had to be excluded from the analysis due to poor DNA quality, low DNA amount or limited tissue availability. All samples were fixed in neutral-buffered formalin prior to paraffin embedding (FFPE-samples). Tumor areas were selected by a senior pathologist (E.W.) on a hematoxylin-eosin stained slide and DNA was extracted from six corresponding unstained 10 μm thick slides by manual macro-dissection. The DNA isolation was performed automatically using the BioRobot M48 (Qiagen, Hilden, GER) as described before [26].

### Sanger sequencing

Exon 4–8 of the *TP53* gene (transcript ID: ENST00000269305, Ensemble Genome Browser, Oct. 2012, http://www.ensembl.org/index.html, [27]) were amplified by PCR using specific primers. *TP53* exon 4 was divided into two PCR reactions due to the exon size (4a and 4b). Primer sequences are listed in S2 Table.

5 μl of PCR products were purified with exonuclease I and Fast-AP (Thermo Fisher Scientific, Waltham, USA) for 15 min at 37°C and 15 min by 80°C. A sequencing reaction was set up with 1 or 2 μl of purified PCR products depending on the amount of PCR products and the BigDye^®^ Terminator v1.1 Cycle Sequencing Kit (Thermo Fisher Scientific Inc.) following the manufacturer’s instructions. The BigDyeXTerminator^®^ Purification Kit (Thermo Fisher Scientific Inc.) was used for the purification of the DNA sequencing reactions removing non-incorporated BigDye^®^ terminators and salts. The solution was incubated for 30 min with agitation of 1800 rpm. The sequencing analyses were carried out on the 3500 Genetic Analyzer (Thermo Fisher Scientific Inc.). The electropherograms were analyzed by visual inspection and Sanger sequencing was repeated by an uncertain or mutated result.

### Statistical analysis

The results of the immunohistochemical and mutation analyses were merged with the clinical-pathological data. The data from the population to be evaluated was analyzed from February 4, 2004, through December 31, 2013, using SPSS statistical software, version 22.0 (SPSS Inc) as described before [28]. The recurrence-free survival (RFS) was calculated using the Kaplan-Meier method from the date of randomization to the date of GIST recurrence or death, censoring patients alive and without recurrence on the date of the last follow-up. We compared the survival rate between groups using a multivariate survival Cox proportional hazards model.

## Results

### Correlation of the expression of eight proteins with recurrence free survival in primary GIST

Expression of p16 (CDKN2A), cyclin D1 (CCND1), p53 (TP53), p21 (CDKN1A), E2F1, CDK4, MDM2 and p-RB1 expression are depicted in Table 1 and S3 Table.

**Table 1:** Association of expression of 8 proteins in primary GIST with recurrence-free survival in the SSGXVIII trial.

Factor	Cut-off (median)	No. of tumours (%)	5-year RFS (%)	p^#^
p16 (CDKN2A), mean	≤2 vs.	178 (55.6)	60.4	
	>2	142 (44.4)	52.2	0.320
Cyclin D1 (CCDN1), mean	0 vs.	178 (55.6)	55.0	
	>0	142 (44.4)	59.3	0.170
p53 (TP53), mean	≤2 vs.	207 (64.7)	62.4	
	>2	113 (35.3)	45.9	0.012*
MDM2, mean	0 vs.	281 (87.8)	58.7	
	>0	39 (12.2)	46.7	0.060
CDK4, mean	0 vs.	215 (67.2)	52.2	
	>0	105 (32.8)	67.6	0.014^+^
p-RB1, mean	0 vs.	241 (75.3)	56.5	
	>0	79 (24.7)	57.8	0.605
p21 (CDKN1A), mean	≤3 vs.	185 (57.8)	59.0	
	>3	135 (42.2)	53.5	0.605
E2F1, mean	≤2 vs.	167 (52.2)	60.0	
	>2	153 (57.8)	53.2	0.234

^#^ determined by log-rank test, p < 0.05 was defined as significance,

* Expression of TP53 is associated with an unfavorable recurrence-free survival,

^+^ CDK4 expression was associated with a better recurrence-free survival

Among the samples of 157 females (49.1%) and 163 males (50.9%) with a median age of 61 years (range 23–83) we could detect a significant increase of p53 (TP53) expression with age (p = 0.015, Mann-Whitney test). With increasing tumor size (median primary tumor size 9.4 cm; range 1.5–35.0) we observed a loss of cyclin D1 (CCND1, p = 0.040) and p21 (CDKN1A, p = 0.037, Mann-Whitney test). No further significant association of cell cycle regulating protein expression was found with gender, tumor site and mutational status.

Concerning the mitotic count, a median of 6 mitoses per 50 HPF could be detected (range 0–135). GISTs with higher mitotic counts showed a significant increased expression of cyclin D1 (CCND1), p53 (TP53) and E2F1 (p = 0.001, <0.001 and <0.001, respectively) whereas elevated levels of p21 (CDKN1A), p16 (CDKN2A) and p-RB1 were found, these factors did not reach significance. 167 (52.2%) of the tumors were localized in the gastric wall whereas 151 (47.5%) were localized elsewhere. The expression of p16 (CDKN2A) and E2F1 significantly correlated to a non-gastric localization (p = <0.001 and p = 0.008 respectively, chi square test). A significantly higher expression of p21 (CDKN1A, p = 0.009) and E2F1 (p = 0.022) could be detected in *KIT* mutated GISTs compared to *PDGFRA* mutated and wt GISTs whereas no significance was reached for the other proteins.

**Fig 1:**
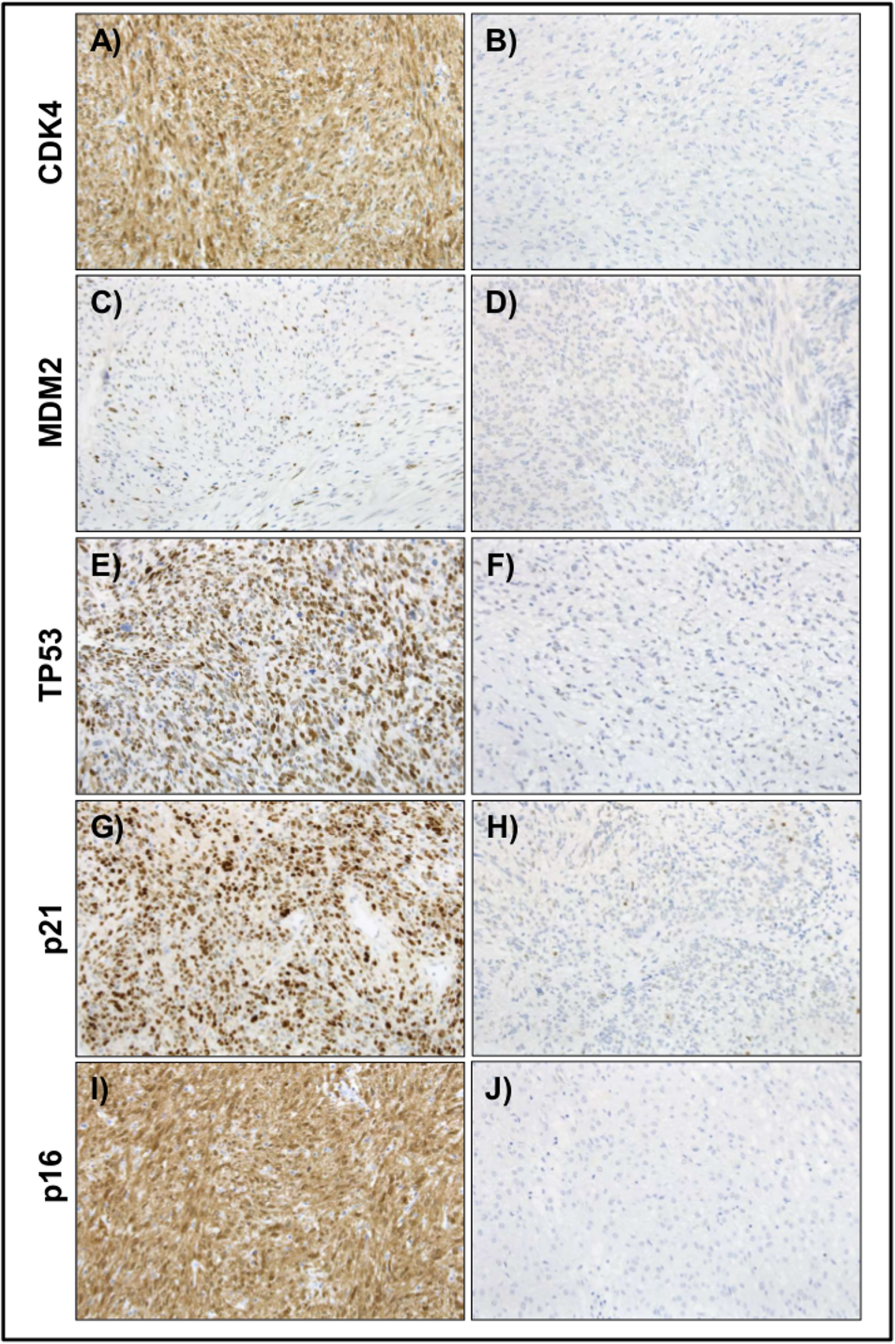
Representative immunohistochemical stainings of CDK4, MDM2,p53, p21 and p16 expression. The expression of different proteins was evaluated by immunohistochemistry. In (A), (C), (E), (G) and (I) the representative high expression pattern of CDK4, MDM2 and p53 (TP53) in different GISTs are shown in contrast to the non/low expression patterns (B), (D), (F), (H) and (J) (100x).

**Fig 2:**
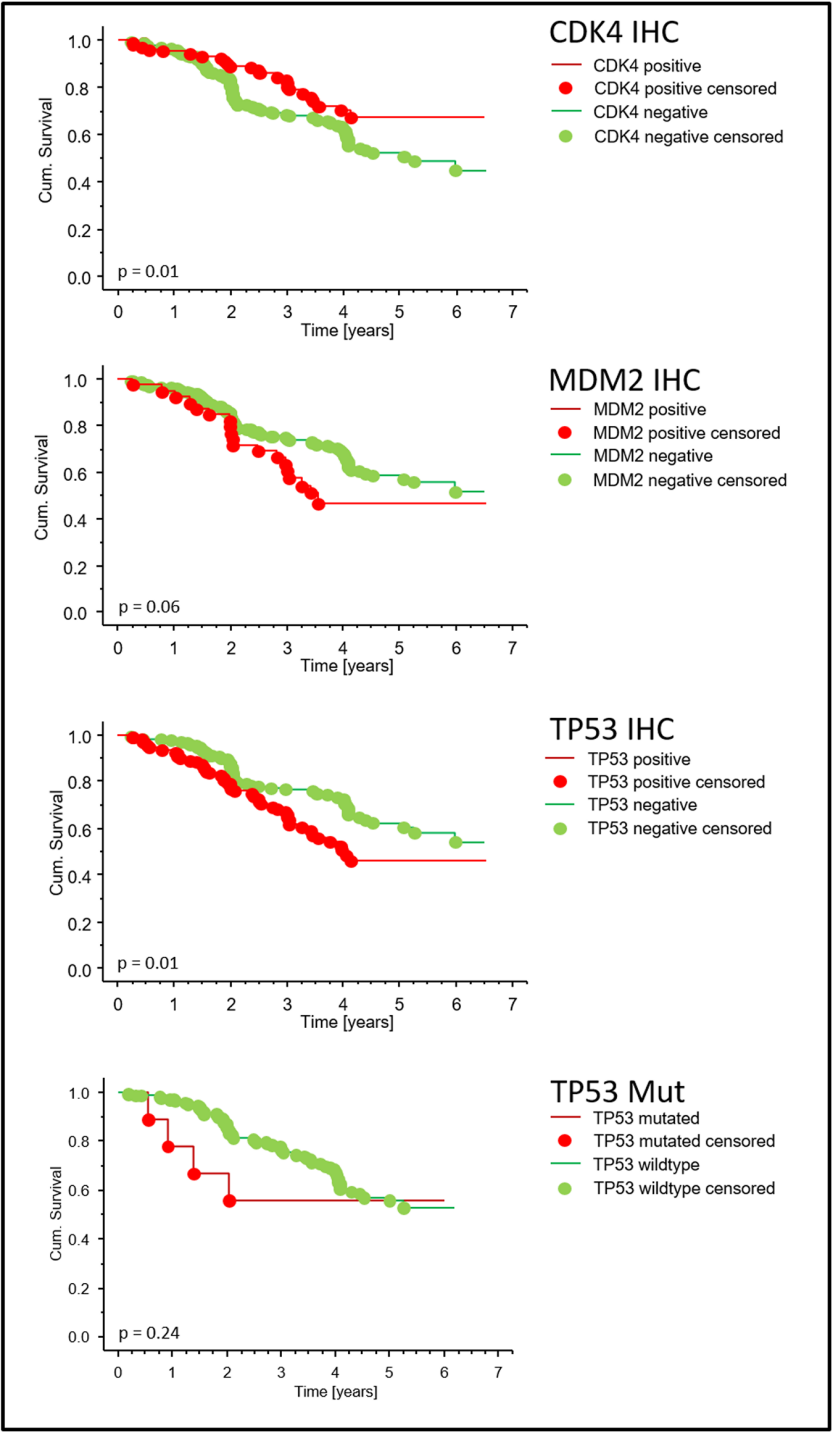
Recurrence free survival of patients with CDK4, MDM2 and p53 expression or *TP53* mutated tumors. Kaplan-Meier survival curve showing analysis of recurrence free survival in patients from the SSGXVIII study. A high expression of CDK4 is positively correlated with recurrence free survival (red line, p = 0.01) in contrast to a high expression of MDM2 and p53 (TP53, red line, p = 0.06 and p = 0.01, respectively, log-rank test). *TP53* mutations tend to correlate to a worse recurrence free survival but did not reach significant levels due to a small number of mutated tumors (p = 0.24, log-rank test).

Concerning the RFS, GIST with high p53 (TP53) expression showed a significant lower five years RFS (45.9%) than those with a low p53 expression (62.4%, p = 0.012, logrank test, Figs 1 and 2).

Similar results were obtained for the expression of E2F1. 243 samples were negative for the expression of E2F1 whereas 77 were strongly positive correlating with a shorter RFS (45.3% versus 60.7%; p = 0.027, log-rank test). Surprisingly, the expression of CDK4, observed in 105 cases, was associated with a longer RFS (p = 0.014, log-rank test, Figs 1 and 2).

**Fig 3:**
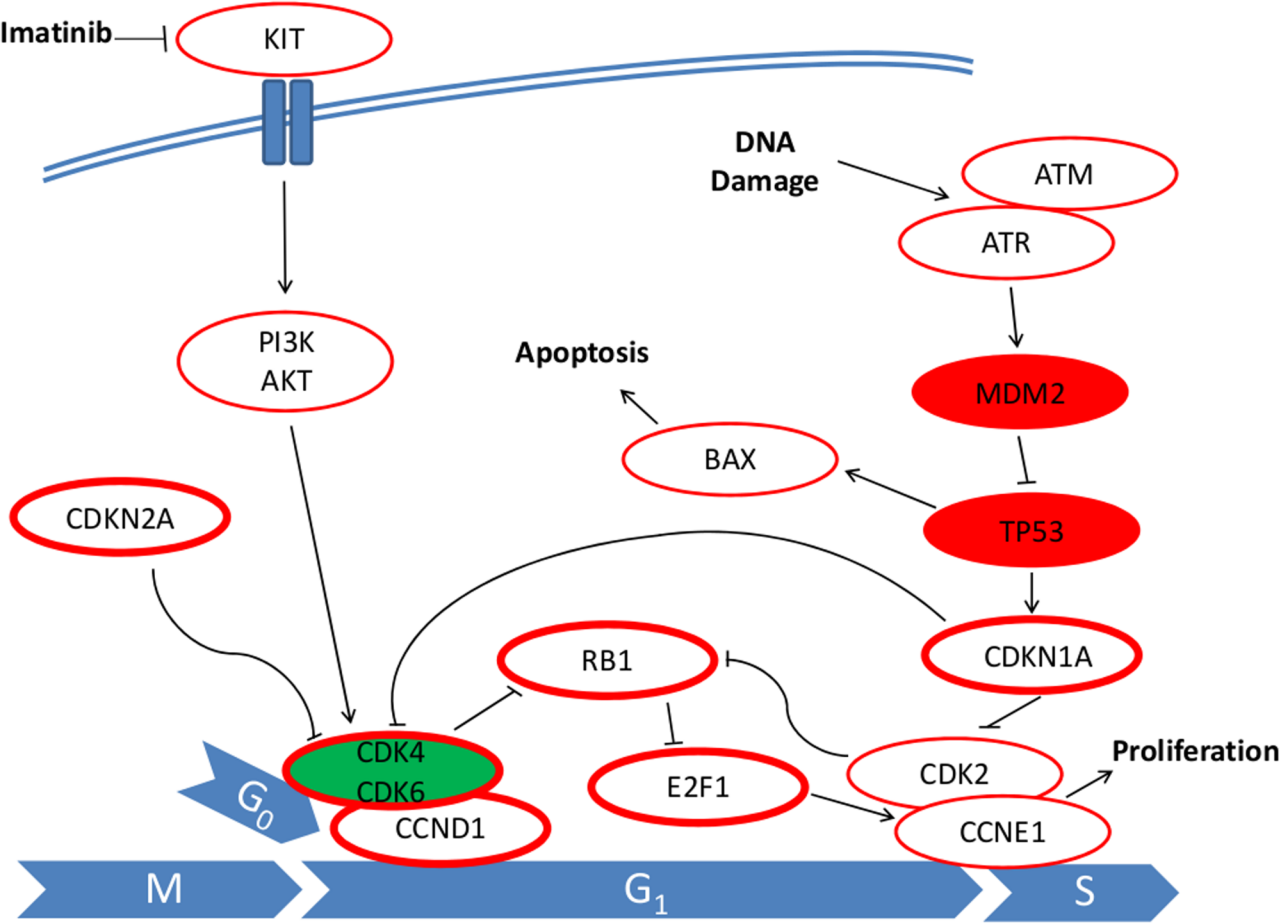
Model of cell cycle regulators and apoptosis modulators that are associated with impaired p53 (TP53) function in GIST. Constitutive activated KIT or PDGFRA promotes proliferation and can be inhibited by imatinib. Impaired p53 function leads as well to a higher proliferation and inhibits apoptosis by modulation of cell cycle regulators. The analyzed proteins are displayed as circles with a bold boarder. Filled green circles indicate that this protein is associated with a favorable recurrence free survival in our study, filled red circles indicate that these regulators are associated with an unfavorable recurrence free survival.

No significant correlation of RFS and the expression of cyclin D1 (CCND1, p = 0.170), p21 (CDKN1A, p = 0.505), p16 (CDKN2A, p = 0.320), MDM2 (p = 0.060) and pRB1 (p = 0.605) could be observed. In summary, it can be stated that although the high expression of p53 (TP53) and E2F1 was significantly associated with a shorter RFS and the expression of CDK4 with a favorable RFS, in the multivariate survival analysis no independent prognostic value for any of the cell cycle regulators could be determined. The result remained the same when the duration of adjuvant imatinib (either for 1 year or for 3 years) was added as a covariable to the model. The main immunohistochemical findings of this study are displayed in Table 2 and a model of cell cycle regulators and apoptosis modulators in Fig 3.

**Table 2:** Main immunohistochemical findings of this study.

Protein	Cases with high expression [%]	Correlation with clinico-pathological parameters	predicitve value
cyclin D1 (CCDN1)	44.4	tumor site, mitotic count	-
p21 (CDKN1A)	42.2	*KIT* mutation	-
p16 (CDKN2A)	44.4	tumor site	-
CDK4	32.8	-	Better RFS
E2F1	47.8	mitotic count, tumor site, *KIT* mutation	-
MDM2	12.2	-	Worse RFS
p53 (TP53)	35.3	age, mitotic count	Worse RFS
p-RB1	24.7	-	-

### *TP53* mutation frequency in GISTs

**Fig 4:**
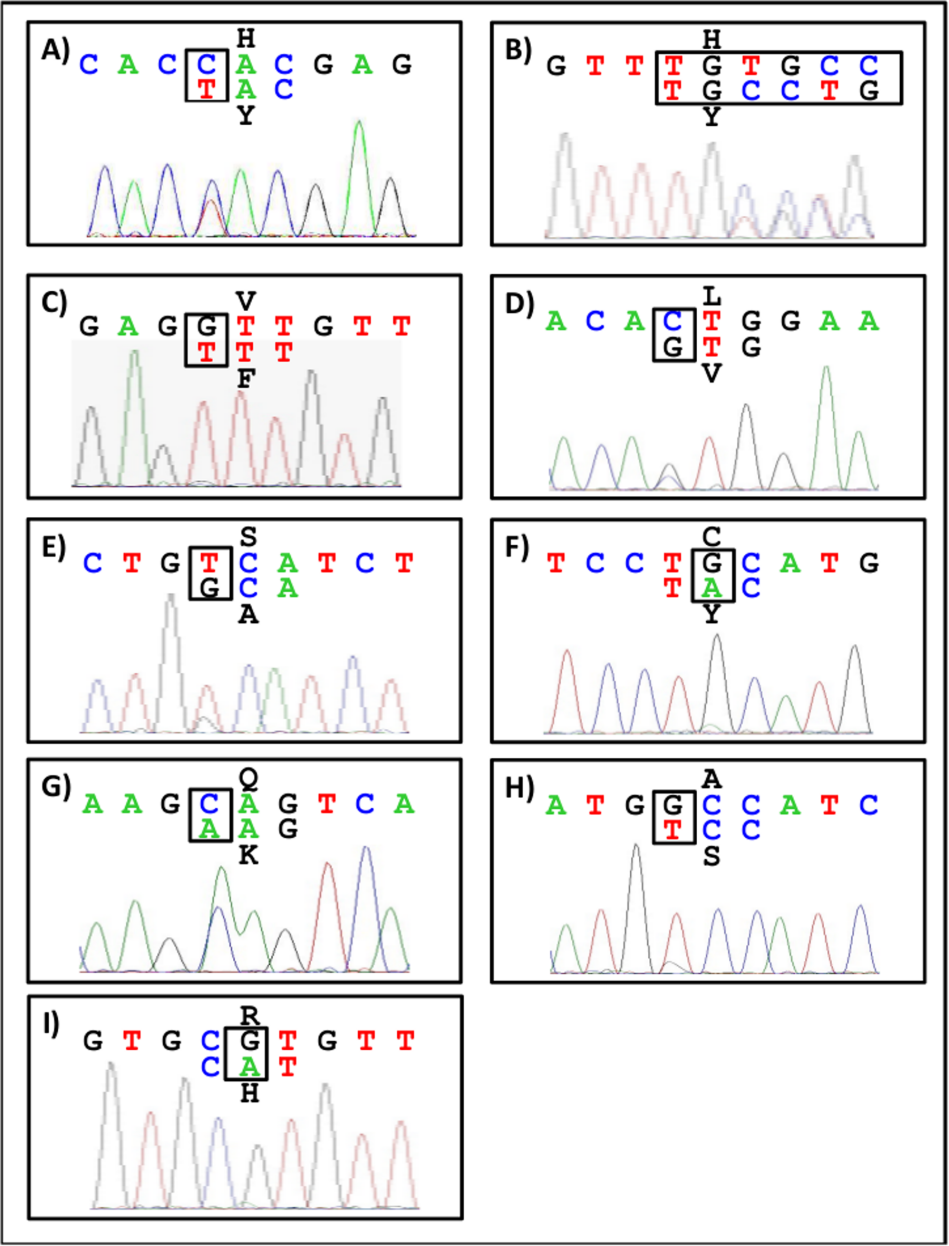
Representative electropherograms of *TP53* mutated GIST samples. *TP53* mutations were detected by Sanger sequencing. Representative electropherograms are shown for each *TP53* mutated sample (A-I) in the same order as shown in Table 1. Six mutations resulted in a non-functional p53 (A-D, F, I), two mutations resulted in a partially functional p53 (G-H) and one mutation could be detected without influence on the p53 function (E).

Nine of the 245 analyzed GIST samples exhibited a mutation in the *TP53* gene (3.5%, Table 3, Fig 4). No double mutations could be detected and only one case showed the *TP53* mutation in a low allelic frequency (case 9). Six of these mutations resulted in a non-functional protein, two in a partially functional protein and one had no influence on the protein function [29]. A closer look at the clinical-pathological findings reveals that eight of the nine *TP53* mutated GIST were suitable for mutation analysis of *KIT* and *PDGFRA*. Four cases harbored a *KIT* exon 11, one sample a *KIT* exon 13, one case a *PDGFRA* mutation and one showed a wildtype status for the two genes. Seven of the 167 gastric samples (52.2% of the whole cohort) showed a *TP53* mutation (4.2%). The other two *TP53* mutations were detected in a GIST localized in the colon and another in a small intestinal GIST.

**Table 3:** Clinical and histopathological findings of GIST with TP53 mutations.

Case No.	Age/Gender	Localization	Size (cm)	Morphological subtype	*KIT/PDGFRA* mutation	*TP53* mutation (effect on protein^#^)	TP53 (p53) expression	Follow up
1	60/F	Stomach	5.5	Epitheloid	*PDGFRA* exon 18: p.I843_D846del	Exon 8: p.H297Y (non-functional)	Non/low	36 Mo; NED** 9.0 yrs
2	67/M	Stomach	13.0	Spindle	*KIT* exon 13: p.K642E	Exon 8: p.A276Lfs*29 (non-functional)	high	12 Mo; rec. 1.4 yrs, DOD^§^ 4 yrs
3	66/M	Stomach	6.0	Epitheloid leiomyomatous	Wildtype^~^	Exon 5: p.V172F (non-functional)	high	12 Mo; rec. 0.6 yrs, DOD^§^ 1 yrs
4	54/F	Colon	5.0	Spindle	*KIT* exon 11: p.K550_Q556delinsM	Exon 7: p.L257V (non-functional)	high	12 Mo; rec. 5.7 yrs
5	56/F	Stomach	6.0	Mixed	*PDGFRA* exon 18: p.D842V	Exon 4b: p.S94A (functional)	high	36 Mo; rec. 0.9 yrs
6	26/F	Small intestine	15.0	Spindle	*KIT* exon 11: p.V559D	Exon 7: p.C242Y (non-functional)	Non/low	36 Mo; rec. 3.2 yrs
7	71/F	Stomach	10.5	Spindle	*KIT* exon 11: p.K550_Q556del	Exon 5: p.Q165K (partially functional)	Non/low	36 MO; NED 5.9 yrs
8	61/M	Stomach	3.0	Epitheloid	*KIT* exon 11: p.L576P	Exon 5: p.A161S (partially functional)	Non/low	12 Mo; rec. 2.0 yrs
9	55/M	Stomach	11.0	Spindle	NA	Exon8: p.R273H (non-functional)	high	36 Mo; NED 5.2 yrs

NA, not available.

^#^ According to the International Agency for Research on Cancer (IARC) TP53 database [29].

^§^ DOD, death of disease

** NED, no evidence of disease

^**~**^ immunohistochemically no loss of expression of succinate dehydrogenase subunit B (SDHB) was detected

Although no significant correlation was observed to tumor size, morphological subtype, risk classification, localization and *KIT*/*PDGFRA* mutation, it is interesting to note that seven of the nine *TP53* mutated GIST were localized in the stomach including the one case with a *TP53* mutation not altering the protein function. Although gastric GISTs are thought to behave less aggressive than intestinal GISTs they were overrepresented among the *TP53* mutant GISTs. Therefore we conclude that *TP53* mutations tend to occur more frequently in gastric GISTs associated with a worse RFS.

## Discussion

In this study, we demonstrated that a high expression of CDK4 was surprisingly associated with a favorable recurrence free survival (RFS), whereas a high expression of MDM2 or p53 (TP53) was associated with unfavorable RFS. These results were independent from the underlying primary *KIT* or *PDGFRA* mutation.

High p53 (TP53) expression is related to an increased half-life time of the mutated protein [30, 31] Here, we showed that 7.1% (n = 2) of the 28 tumors with a high p53 (TP53) expression showed a mutation in the *TP53* gene. The screening of the whole cohort for *TP53* mutations revealed an overall frequency of 3.5%. This is in concordance with other studies showing a very low *TP53* mutation frequency despite a high TP53 protein expression in a higher number of GIST [11, 32–35]. One explanation of this mutation independent activation of p53 (TP53) might be amplification or posttranscriptional modifications such as phosphorylation, acetylation or sumoylation [36, 37].

By correlating the *TP53* status with clinico-pathological parameters, we demonstrated that *TP53* mutations tend to correlate with a poor RFS. Nevertheless, the results did not reached significance. Obviously, *TP53* mutations are a rather late event in GIST progression as the percentage of *TP53* mutated GISTs was higher in the study of Romeo *et al*. in a metastatic GIST cohort and in a study of Ryu *et al*. in a series of 125 localized GIST with 22% *TP53* mutated tumors. Interestingly, both studies preselected the tumor samples for mutation analysis depending on their immunohistochemical p53 (TP53) expression which is, according to our results, not a sensitive biomarker for the TP53 genotype.

Others have shown that *TP53* mutations are necessary for the malignant progression of breast cancer from lower to higher grades [38] and that these mutations therefore significantly correlate to a higher mitotic count and a higher risk group in GIST [34]. In ovarian cancer *TP53* mutations significantly correlate to resistance to platinum-based chemotherapy and shortened survival [39]. The same tendency could be observed in supratentorial WHO Grade II astrocytomas and oligoastrocytomas [40] where *TP53* mutations were unfavorable predictors of progression free survival. In our cohort the absolute number of *TP53* mutations in GIST is too low to draw final conclusions. However, determining the *TP53* mutational status may help to identify patients with a relatively high risk of recurrence prior to treatment and therefore the *TP53* status should be considered to select patients who will best benefit from an adjuvant treatment.

Furthermore, we could detect a significant correlation of high expression of MDM2 with poor RFS. This is in concordance with the current literature. High MDM2 expression is correlated with poor progression free survival in ovarian clear cell carcinoma [41] and with poor overall survival in urothelial neoplasms of the bladder [42]. In GIST a high mRNA expression of MDM2 is described with an aggressive clinical behavior and an unfavorable prognosis analyzing 38 GIST samples of all risk classifications [43].

We also detected a high expression of CDK4 in 32.8% of the cases. High expression of CDK4 is frequently seen in human cancer [44]. Dai *et al*., analyzing triple negative breast cancer, described a high expression of CDK4 correlating with poor overall survival and relapse free survival [45]. In contrast, Peurala *et al*., found no correlation of CDK4 expression with survival data in human breast cancer [44]. An upregulation of CDK4 is described in 85% of myxoid and round cell liposarcoma [46]. Furthermore, a high level of CDK4 amplification is a poor prognostic marker in well-differentiated and dedifferentiated liposarcoma [47]. In GIST, Sabah *et al*. detected CDK4 expression in 100% of the samples but without an association between CDKs and histological classification [48].

CDK4 is an important regulator of the cell cycle G1 phase progression and the G1 to S phase transition. In detail, CDK4 phosphorylates RB1 protein, lead to the inactivation of RB1 and the dissociation from E2F1 transcription factor [49]. RB1 is a tumor suppressor that undergoes periodic phosphorylation when cells transverse the cell cycle [50]. RB1 is dephosphorylated when cells exit the mitosis and phosphorylated in late G1 phase and remains so throughout the progression through S phase to mitosis. This results in the activation of multiple genes encoding proteins that are required for DNA synthesis or to enter the S phase.

Therefore, the activated, hypophosphorylated RB1 protein restricts proliferation [51]. By phosphorylation and therefore inactivation of RB1, CDK4 is an essential regulator of proliferation. CDK4 is crucial for various oncogenic transformation processes suggesting that many cancer cells may be addicted to high CDK4 activity [44]. However, we detected a significant correlation of CDK4 expression with a favorable RFS. This is in contrast to the current literature and in contrast to the findings expected from the biological function of CDK4. One explanation might be the unique cohort of well-characterized high risk GIST prior to treatment and the high number of samples analyzed. To our knowledge, this is the only study analyzing more than 200 high risk GIST prior to treatment for the expression of cell cycle regulators.

Furthermore, we observed a significantly higher expression of E2F1 in *KIT* mutated GISTs compared to *PDGFRA* mutated and wt GISTs, in tumors with non-gastric location and with higher mitotic counts. Similarly, expression of p16 (CDKN2A) was upregulated in *KIT* compared to *PDGFRA* mutated and wt GISTs and associated with a non-gastric location. GISTs with higher mitotic counts showed a significantly increased expression of cyclin D1 (CCND1) and p53 (TP53). These results are in concordance with other studies [43, 52, 53]. A comparable p53 (TP53) expression profile was described in 25–38% of GISTs by different groups [34, 54–56]. In a study of Romero *et al*., 2009, which analyzed samples from patients with advanced metastasized GIST and which tested 400 mg versus 800 mg imatinib daily [25], proved that tumors had a higher expression of p16 (CDKN2A, 57%) and of p53 (TP53, 47%), whereas expression of p21 (CDKN1A, 27%) and cyclin D1 (CCND1, 8%) was lower. Their expression data of MDM2 (in 13%) and CDK4 (in 31%) were comparable to our findings. The higher frequency of p53 (TP53) immunohistochemical positive samples in their cohort of metastatic GISTs may reflect the more advanced stage of disease compared to our cohort of still localized non-metastatic but high risk GISTs prior to adjuvant imatinib treatment. In another study, Haller *et al*. showed that GISTs with a *KIT* exon 11 deletion exhibited a higher cyclin D1 (CCND1) expression that correlated with an accelerated proliferation and shorter disease free survival in untreated primary GISTs and that high mRNA expression of CDK4, p16 (CDKN2A), RB1, MDM2, p53 (TP53) and E2F1 correlated with poor prognosis [43, 57]. Surprisingly, we detected a marginal significance between the lack of CCND1 (cyclin D1) expression and larger tumors. This is likely due to the SSGXVIII trial inclusion criteria, since patients with a large GIST (>10 cm in diameter) were considered high risk GISTs regardless of the tumor mitotic count (according to the modified NIH risk stratification scheme). Therefore, many large GISTs with a small mitotic count were included in the trial, which explains the marginally significant association between absence of CCND1 expression and a small tumor diameter.

In our study on formalin-fixed, paraffin-embedded tissue, p16 (CDKN2A) showed no significant correlation with RFS. This discrepancy might be due to the use of different material. However, we detected a correlation of p16 (CDKN2A) expression with non-gastric localization. Extra-gastrointestinal stromal tumors (E-GIST) show aggressive clinico-pathological parameters as shown for hepatic E-GIST (high mitotic counts: >5/50 HPF, large tumor size: >5 cm) resulting in a high risk classification [58, 59]. Therefore, we could indirectly show that the expression of p16 (CDKN2A) correlated with a worse prognosis.

In summary, although none of the eight histological parameters was independently associated with recurrence-free survival (RFS) in a multivariable analysis with the standard prognostic factors, we demonstrated an increased expression of several cell cycle regulating proteins in GIST. The result remained the same when the duration of adjuvant imatinib (either for 1 year or for 3 years) was added as a covariable to the model.

Our study suggests that the expression of MDM2, CDK4 and p53 (TP53) and the mutational status of *TP53* may be useful to identify patients with high risk localized GIST who carry an increased recurrence risk and may benefit from a prolonged adjuvant treatment with imatinib although the *TP53* mutation frequency is very low in treatment naïve, high risk GIST.

## Supporting information

S1 Table Antibodies. (XLSX)

S2 Table Primer sequences. (XLSX)

S3 Table Laboratory assay results. (XLS)
